# Influence of Printing Orientation on Tensile Strength and Surface Characterization of a Steel-Powder-Reinforced Thermoplastic Composite Manufactured by FDM Technology

**DOI:** 10.3390/ma18245656

**Published:** 2025-12-17

**Authors:** Paweł Szczygieł, Krystyna Radoń-Kobus

**Affiliations:** Department of Mechatronics and Mechanical Engineering, Kielce University of Technology, al. Tysiąclecia Państwa Polskiego 7, 25-314 Kielce, Poland; kradonkobus@tu.kielce.pl

**Keywords:** 3D printing, tensile strength, wettability

## Abstract

This study presents results on a thermoplastic polymer composite containing 95 wt.% steel powder, processed into samples using Fused Deposition Modeling (FDM) technology. Such a high filler loading exceeds the values commonly reported in the literature. Samples were printed with different build orientations (0° and 90°) to evaluate the influence of printing direction on the tensile behavior of the material. Tensile tests were conducted to determine the effect of printing orientation on the composite’s strength. Additionally, surface structure analysis was performed using a contact profilometer, and wettability was evaluated by measuring the contact angle with a tensiometer. Dimensional measurements were also carried out using a digital caliper. The obtained results allowed determination of the relationship between printing orientation and the tensile and surface-related properties of the analyzed composite material.

## 1. Introduction

One of the additive manufacturing (AM) methods is material extrusion, known as Fused Deposition Modeling (FDM), which is among the most widely used and rapidly developing 3D printing techniques. The feedstock material is typically a thermoplastic filament (a strand of specified diameter wound on a spool), which can be either a pure polymer or a composite. Polymer composites are formed by combining at least two materials, enabling the production of a material with enhanced mechanical and functional properties, such as thermal or electrical characteristics. In a typical composite, one component serves as the reinforcing phase, while the other acts as the matrix. In FDM, various types of reinforcement are used, including particles, fibers, or nanomaterials, allowing for tailoring of desired properties. The most common are composites reinforced with micro- or nanoparticles, metal particles, or short or continuous fibers [1]. The addition of metals (iron, copper, stainless steel, titanium) or nanoparticles (carbon nanotubes, graphene, graphite) enables the production of materials with increased strength, dimensional stability, and thermal and electrical conductivity [2].

The authors [3] demonstrated that FDM printing parameters, particularly print orientation, have a significant impact on the mechanical properties of 3D-printed PLA composites reinforced with bronze particles. Parts printed in the “On-Edge” orientation (sample positioned on its side) exhibited the highest tensile stresses, whereas samples printed in the vertical (“Upright”) orientation were highly brittle. In study [4], the researchers showed that reinforcing PLA filament (polylactic acid) with 5% iron powder significantly improves its mechanical properties, increasing both tensile strength and hardness, although it also raises surface roughness. However, pure iron can be prone to oxidation and corrosion over time, which may reduce material durability and cause degradation under environmental conditions.

The build orientation in additive manufacturing technologies strongly influences surface texture, as it determines the layer deposition pattern and the need for support structures. In FDM, three types of surfaces with distinctly different roughness can be identified: top-layer surfaces, which exhibit the lowest roughness (Ra ≈ 3.77 µm); support-adjacent surfaces, with higher Ra values (≈9.47 µm) that often require post-processing; and layer-type surfaces, where the deposited layers are clearly visible and which show the highest roughness (Ra ≈ 14.07 µm) [5]. Studies on PLA FDM indicate that increasing the print orientation angle generally increases Ra due to the amplification of the stair-stepping effect, whereas, at very high angles (80–90°), a decrease in roughness is observed due to surface profile smoothing [6]. In powder-based technologies such as LB-PBF/SLM, orientation also significantly affects surface quality, with the lowest 2D and 3D roughness parameters typically obtained at 0° orientation, parallel to the build platform [7]. Similar trends were reported for titanium (cp-TiGd2), where horizontally printed samples showed the lowest roughness, while 45° orientation generated surfaces with the highest Ra and Rz values [8].

The state of surface geometry is closely related to its wettability. The contact angle (CA) is a physical parameter used to assess the interaction between a solid surface and a liquid and serves as a key measure of surface wettability. The most commonly used method is the measurement of the static contact angle, in which the droplet remains stationary, and its volume does not change, typically using the sessile drop technique [9]. Hydrophilicity and hydrophobicity are basic categories describing surface wettability depending on the extent to which a liquid spreads on a surface. A static contact angle (CA) below 90° indicates hydrophilicity, meaning the surface is easily wetted by the liquid. A CA above 90° indicates hydrophobicity, where the surface is difficult to wet, and a CA greater than 150° classifies the surface as superhydrophobic, exhibiting strong resistance to wetting [10]. Build orientation, defined as the positioning of the part on the 3D printer platform and the arrangement of layers in FDM technology, significantly affects the contact angle of printed surfaces. Surfaces parallel to the layers (0°) generally exhibit lower and more uniform contact angles, whereas surfaces perpendicular to the layers (90°) show a marked increase in CA due to the stair-step and layered structure formed during printing. Changing the layer orientation increases the anisotropy of the contact angle and may lead to partial wetting of the surface. Materials used in FDM, such as pure PLA, are generally hydrophilic, while 15% CF-PLA composites, modified with carbon fiber, exhibit higher contact angles, favoring more hydrophobic surfaces [11,12].

Print orientation is also critical for mechanical properties, particularly tensile and flexural strength. The build direction of a part (print orientation) has a significant impact on its strength and other mechanical characteristics, which directly reflects anisotropy (the phenomenon in which a material’s properties vary depending on the direction). This anisotropy primarily arises from the layered nature of AM techniques, which produce different structures and defects depending on how the material layers are deposited and bonded. In FDM technology, print orientation strongly influences the mechanical performance of printed samples [13]. For PLA, changing the part orientation from flat to vertical was shown to reduce tensile strength by up to 36%, due to interlayer fracture [14]. In the case of ABS (acrylonitrile butadiene styrene) and ULTEM (polyetherimide), the best mechanical properties were achieved when the layers were aligned parallel to the applied tensile force, whereas vertical orientations promoted interlayer separation and reduced strength [15,16].

In the study by [17], FDM composites based on PLA, carbon fiber-reinforced PLA (CF-PLA), and other polymer filaments were analyzed. The results showed that PLA composites reinforced with carbon fibers or other structural fillers exhibited significant improvements in tensile, bending, and interlayer shear strength, while metal additives such as aluminum enhanced interlayer bonding and thermal properties. However, for FDM composites containing metallic or ceramic fillers, reported filler contents typically do not exceed 65 vol.% due to processing limitations and reduced melt flow [18]. The present work investigates a thermoplastic composite with 95 wt.% steel powder, which is rarely addressed in the literature. This high filler content is technologically relevant, as it enables polymer–metal structures with improved stiffness, density, and functional properties while introducing challenges in printability, interlayer bonding, and surface wettability. The novelty of this study lies in evaluating how build orientation (0° and 90° relative to the build platform) affects these properties in a high-filler FDM composite, addressing the gap in knowledge on highly steek-filled filaments where comprehensive studies of surface topography, mechanical performance, and wettability are currently lacking.

This study aims to evaluate the influence of build orientation on the geometric structure, surface wettability, and tensile strength of a thermoplastic composite reinforced with steel powder, printed using FDM. The results demonstrate how steel powder modifies these properties depending on layer orientation, confirming the rationale for this type of reinforcement. Furthermore, the obtained data can serve as guidance for teams designing and optimizing powder-reinforced composites to achieve improved functional and mechanical performance.

## 2. Materials and Methods

The samples were fabricated from a material commercially known as AIB METAL (AIB, Knurów, Poland). According to the manufacturer, parts printed with this filament resemble steel in appearance, weight, and tactile feel. Additionally, they are characterized by high density and excellent resistance to environmental conditions [19].

The filament is a composite consisting of 316 L steel powder embedded in a non-emitting thermoplastic polymer. According to private correspondence with the manufacturer, the steel content in the material is approximately 95 wt.%. The exact composition of the filament is protected as proprietary *know-how*; however, the material data sheet indicates that the chemical structure includes a mixture of polymers and a metal powder alloy encapsulated within a polymer matrix. The data sheet provides information on the steel powder composition, which is typical for 316 L stainless steel and consists mainly of iron (>60 wt.%), with 16–18% chromium, 10–14% nickel, 2–3% molybdenum, up to 2% manganese, and up to 1% silicon, while the carbon content is low, below 0.03% [20].

The filament manufacturer recommends printing with a nozzle temperature between 235 and 250 °C and on a heated bed at 80 °C, with a print speed of 80–150 mm/s. The filament is gray in color, with a diameter of 1.75 ± 0.05 mm, a density of approximately 4 g/cm^3^, and exhibits good resistance to UV radiation [19].

As mentioned before, the composite filament consists of 316 L steel powder embedded in a proprietary thermoplastic polymer. The exact formulation is not disclosed by the manufacturer; however, based on the extrusion temperature (≈245 °C), lack of pre-drying or closed-chamber requirements, non-emitting nature, and reported UV resistance [19], the polymer can be classified as a high-temperature thermoplastic with properties similar to PETG. This limitation is noted when comparing the results with literature data on other metal-filled filaments.

The material properties are presented in Table 1. According to the manufacturer, the values were obtained from samples produced using the following settings: layer height of 0.12 mm, 100% infill density, linear infill pattern, 3 perimeters, nozzle temperature of 240 °C, and bed temperature of 80 °C [21].

The samples were fabricated from the material in its as-received condition, in the form of a filament with a diameter of 1.75 mm. The 3D models of the samples were designed using SOLIDWORKS 2023 (Dassault Systèmes, Vélizy-Villacoublay, France) and then saved in STL format (Standard Tessellation Language). The additive manufacturing process parameters were set using Bambu Studio software v 2.0.1.50 (Bambu Lab, Shenzhen, China) after importing the STL models.

**Figure 1:**
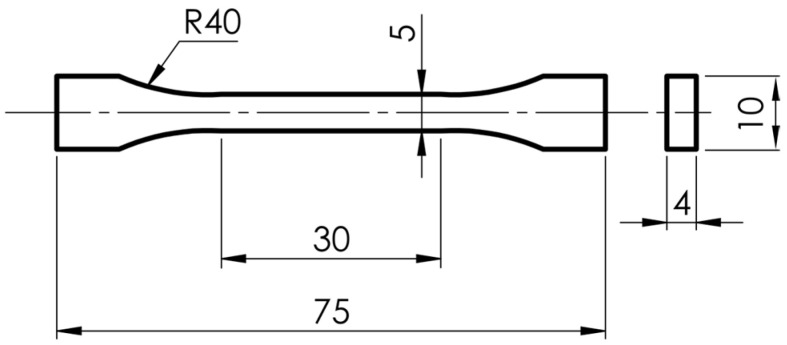
Dimensions of sample S1 (Unit: mm).

The samples were fabricated using a BambuLab X1C 3D printer (Bambu Lab, Shenzhen, China) employing FDM (Fused Deposition Modeling) technology. The printer was equipped with a 0.6 mm hardened steel nozzle and a Textured PEI Plate build surface. Two types of samples were produced depending on their intended use:

(a) S1—samples for surface geometric structure analysis and static tensile testing according to ISO 527 (1BA), as shown in Figure 1;
(b) S2—cylinders with a diameter of 40 mm and a height of 6 mm intended for surface wettability measurements.

The additive manufacturing process parameters are summarized in Table 2. The variable parameter was the build orientation (positioning of the samples on the 3D printer build platform) at two levels: 0° and 90° (the angle between the sample surface and the printer build platform).

**Figure 2:**
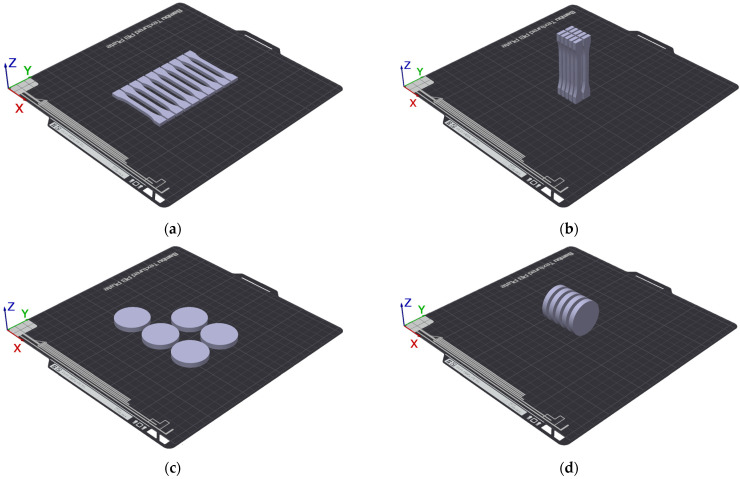
Arrangement of samples on the 3D printer build platform: (**a**) S1-1, (**b**) S1-2, (**c**) S2-1, (**d**) S2-2.

The samples were produced in varying numbers of repetitions, as summarized in Table 3 along with their labels. The arrangement of the samples on the 3D printer build platform is shown in Figure 2.

The fabricated samples were removed from the build platforms. No support structures were used in the manufacturing process. The cross-sectional dimensions of the S1 samples were measured using a digital caliper with a resolution of 0.01 mm and a maximum measurement error of 20 µm over 100 mm (SYLVAC, Yverdon-les-Bains, Switzerland) to enable tensile testing based on the actual sample dimensions. The nominal thickness and width of the samples were 4 mm and 5 mm, respectively. Each sample was measured three times at different points with the caliper to determine the actual dimensions and assess any deviations from the nominal values. The research results are presented in Section 3.1.

Microscopic observations of the S1 samples were performed using a MAHR Vision MN320 digital microscope (Mahr, Göttingen, Germany) with an optical magnification of ×1 under reflected light. The research results are presented in Section 3.2.

Surface topography measurements were performed using a contact profilometer, Form Talysurf PGI 1200, equipped with an environmental chamber (Taylor Hobson, AMETEK, Inc., Berwyn, PA, USA). The stylus had a conical tip. A total of 30 profile measurements were taken for selected tensile test samples with different build orientations. The acquired data were processed using TalyMap Platinum software v 5.1.1.5374 (Taylor Hobson, AMETEK, Inc., Berwyn, PA, USA). No additional filtering was applied, resulting in primary profiles. Each profile was recorded with a step of 0.1 mm over a length of 3 mm. Based on the 30 profiles, an average profile was calculated, which was then used to reconstruct a 3D surface of 3 mm × 3 mm. Surface topography parameters, including Sa and others, were calculated from the reconstructed 3D surface. The research results are presented in Section 3.3.

Static tensile tests were carried out on an Inspekt Mini universal testing machine equipped with LabMaster software v 2.5.3.21 (Hegewald and Peschke, Nossen, Germany). The tests were performed at a crosshead speed of 2 mm/min. The research results are presented in Section 3.4.

Contact angle measurements were performed using an optical tensiometer (Attension Theta Flex, Gothenburg, Sweden) with distilled water as the test liquid. Measurements were conducted on five samples for each build orientation, and averages with standard deviations were calculated. The research results are presented in Section 3.5.

All measurements were carried out under controlled conditions at an ambient temperature of 20 °C and relative humidity of 56%, by a single operator.

The study was supplemented with observations of the surface of sample S2-1 using a Phenom XL scanning electron microscope (Thermo Fisher Scientific, Waltham, MA, USA). The microscope operated at an accelerating voltage of 15 kV and at magnifications of 300× and 5000×. Prior to the observations, the samples were prepared by grinding with abrasive papers of progressively increasing grit sizes: 120, 240, and 600. The research results are presented in Section 3.6.

## 3. Results

### 3.1. Measurement of Sample Cross-Sections

**Figure 3:**
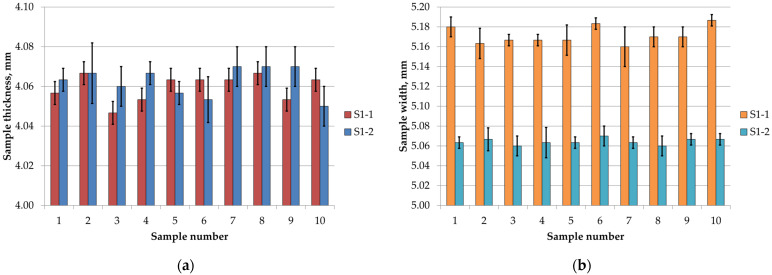
Cross-section measurement results of S1 samples: (**a**) thickness, (**b**) width.

The results of the cross-sectional measurements of the samples are shown in Figure 3, with error bars representing standard deviations. Analysis of the thickness and width measurements for S1-1 and S1-2 series samples indicated high repeatability and minimal deviations from the nominal values.

The nominal thickness of the samples was 4 mm, while the measured values for the S1-1 and S1-2 samples ranged from approximately 4.05 mm to 4.07 mm. These values are slightly higher than the nominal thickness, indicating positive deviations of about 1–2%. Differences between individual samples were small, and the small standard deviations indicate low variability and good repeatability of the measurements.

For the sample width, with a nominal value of 5 mm, a more pronounced difference was observed between the S1-1 and S1-2 series. For the S1-1 series, produced with a 0° build orientation, the values ranged from approximately 5.16 mm to 5.19 mm, whereas for the S1-2 series, produced at 90°, they ranged from 5.06 mm to 5.07 mm. This corresponds to a difference between the two series of about 0.10–0.12 mm, or approximately 2–2.4% relative to the nominal value. The small standard deviations observed for both S1-1 and S1-2 suggest that the measurements were consistent, and the sample dimensions were reproducible.

### 3.2. Microscopic Observations

**Figure 4:**
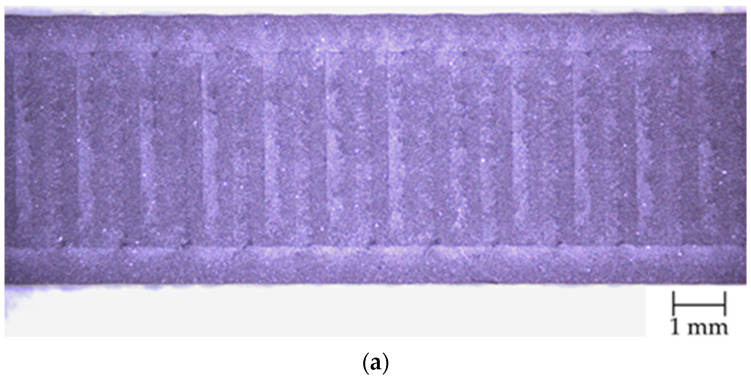

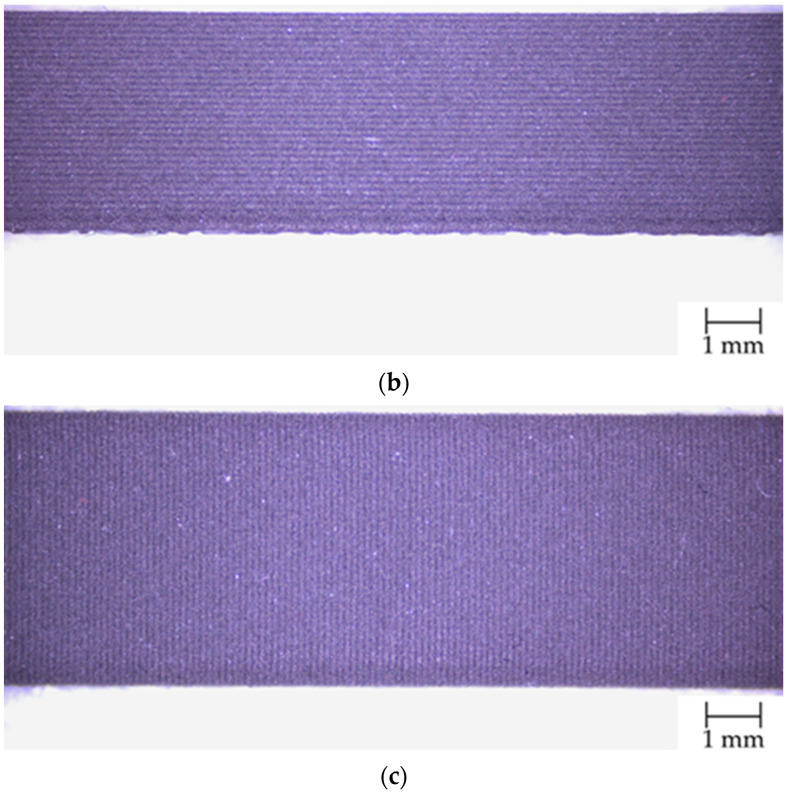
Surface view of samples: (**a**) top S1-1, (**b**) side S1-1, (**c**) side S1-2.

Figure 4 shows the magnified surfaces of samples S1-1 and S1-2 observed using an optical microscope. The top surface of sample S1-1 (Figure 4a) exhibits high regularity and a clearly defined arrangement of parallel deposited lines. The visible paths are relatively wide, which results from the use of a 0.6 mm nozzle during the additive manufacturing process. The surface structure is uniform, and the individual material traces are well-defined, with no observable defects such as voids, microcracks, or edge overheating.

The lateral surface of sample S1-1 (Figure 4b) also exhibits a regular structure, with clearly visible contours of successive deposited layers. The first layer is slightly thicker, which is consistent with the process parameters and intended to improve adhesion to the build plate. The lower part of the sample shows certain irregularities resulting from the use of a Textured PEI Plate, whose surface pattern was partially replicated on the underside of the sample. The boundaries between consecutive layers are generally parallel, although minor local undulations are present, likely caused by small fluctuations in material flow during extrusion. Despite these slight irregularities, no significant surface defects, such as gaps between filaments, under-extrusion, or air inclusions, were observed.

The lateral surface of the S1-2 samples (Figure 4c) exhibits a well-defined layer structure. The lines marking the interfaces between successive layers are straight and parallel, and the layer thickness remains consistent along the entire length of the sample. The edges show a uniform, stepped profile characteristic of FDM-printed components, confirming the proper deposition of successive layers during the manufacturing process.

### 3.3. Contact Profilometer Measurement

**Figure 5:**
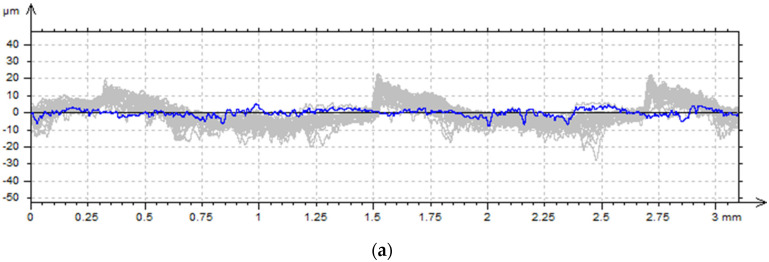

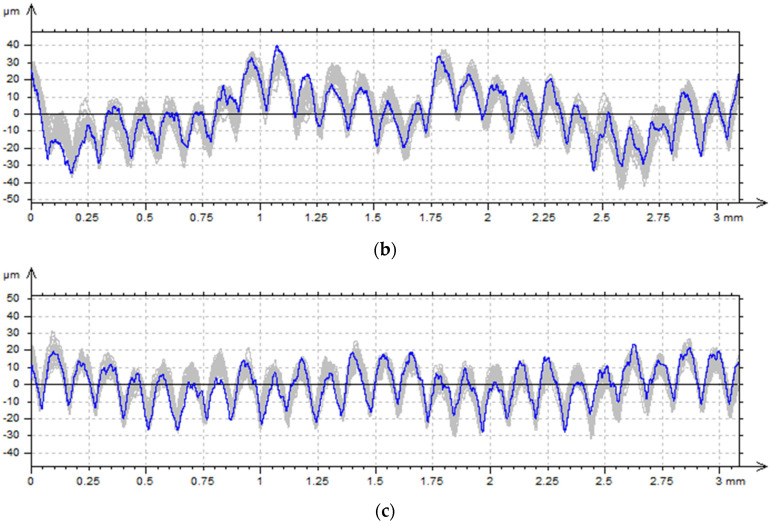
Average primary surface profile of samples: (**a**) top S1-1, (**b**) side S1-1, (**c**) side S1-2.

Analogously to the surfaces previously examined under the optical microscope, surface measurements were performed using a contact profilometer, with the measurement direction oriented perpendicular to the layer-deposition path. Based on the 30 acquired primary profiles, an average profile was calculated for each surface (Figure 5). The grey color represents the obtained primary profiles, whereas the blue color corresponds to the averaged profile. The corresponding profile parameters are summarized in Table 4.

The analysis of the obtained surface topography parameters indicates that the surface characteristics are strongly dependent on the sample orientation during additive manufacturing. For sample S1-1, produced at a 0° orientation, the highest height-related parameters (Pp, Pv, Pz, Pt) were recorded for the side surface. The mean peak height (Pp) was 34.1 µm, while the maximum valley depth (Pv) reached 37.4 µm, resulting in a total profile height (Pz = Pt) of 71.5 µm. These values are nearly twice as high as those obtained for the top surface of the same sample (Pz = 32.96 µm). This indicates a substantially greater amplitude of surface profile irregularities and more pronounced topographical variation on the side surface, which is characteristic of FDM-printed vertical walls, where successive material layers are deposited vertically, forming distinct micro-steps.

For sample S1-2, produced at a 90° orientation, the height parameters were also elevated compared to the top surface S1-1, but lower than those of the side surface of S1-1 (Pz = 51.6 µm). Samples produced at 0° exhibit greater height differences between successive layers.

Comparing the Pa and Pq parameters, which represent the mean height deviation and the root mean square (RMS) height, respectively, a marked increase is observed for the side surfaces. For S1-1 (side), Pa = 11.69 µm and Pq = 14.40 µm, whereas for the top surface the values are 5.09 µm and 6.24 µm, respectively. This indicates that the side walls exhibit more than twice the surface height amplitude. Moreover, the ratio Pq/Pa ≈ 1.23 for both surfaces suggests a similar distribution of profile irregularities.

The parameters Psk and Pku, which describe the height distribution shape, reveal additional differences in microgeometry. The top surface of S1-1 exhibits positive skewness (Psk = 0.347), indicating a predominance of peaks over valleys, likely due to the final filament paths slightly protruding above the plane. For the side surface of S1-1, Psk ≈ 0.05, showing an almost symmetric distribution. In contrast, S1-2 (side) shows negative skewness (Psk = −0.341), suggesting a predominance of valleys resulting from the different layer deposition direction in the 90° orientation. The kurtosis (Pku) values for all surfaces range slightly below 3 (2.54–2.83), indicating a somewhat flattened distribution; the surfaces do not have sharp, isolated peaks but rather smoother, more spread-out irregularities.

Analysis of the Psm parameter, which describes the mean spacing between profile features, corroborates the microscopic observations. For the top surface of S1-1, Psm was 0.394 mm, whereas for the side surfaces it was 0.166 mm (S1-1) and 0.121 mm (S1-2). This indicates that the features on the top surface are considerably more widely spaced, consistent with the use of a 0.6 mm nozzle and the larger distance between consecutive filament paths visible in Figure 4a. In contrast, the lower Psm values on the sides indicate a denser, more uniform structure resulting from the layer deposition pattern. It is worth noting that the samples were produced with a layer height of 0.12 mm (Table 2), so S1-2 accurately reproduces the expected layer structure, whereas the side of S1-1, with a Psm of 0.166 mm, slightly exceeds the theoretical value, suggesting minor deviations in layer replication or an influence of the sample build orientation.

The Pc parameter, which characterizes the core structure of the profile, exhibits higher values on the side surfaces (approximately 31.3 µm for the S1-1 side and 29.9 µm for the S1-2 side) than on the top surface (14.5 µm). This indicates that larger, more regular irregularities dominate the deeper layers of the profile, resulting from the successive deposition of material layers during the FDM process.

Analysis of the standard deviations indicates that the top surface of S1-1 exhibits the highest variability across most amplitude and height distribution parameters. In contrast, the side surfaces of both S1-1 and S1-2 show lower standard deviations, indicating more consistent and uniform microstructure. Notably, the side of S1-2 (90° orientation) demonstrates the smallest variability, particularly in Psm, confirming a highly repeatable layer pattern.

**Figure 6:**
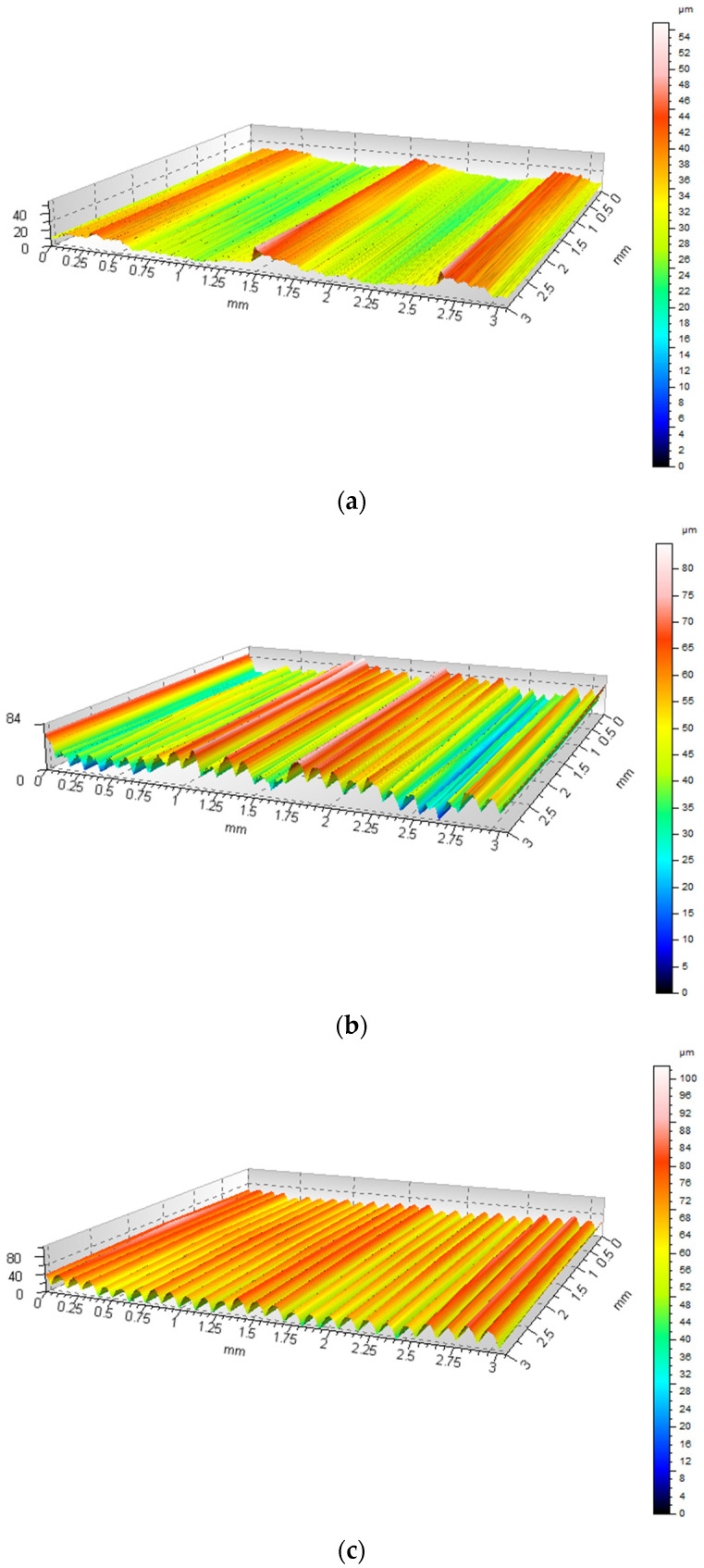
Sample surface topography shown in isometric view: (**a**) top S1-1, (**b**) side S1-1, (**c**) side S1-2.

Based on 30 profiles obtained for each examined surface, a three-dimensional reconstruction of the surface topography was generated. The TalyMap software applied spatial interpolation to fill the areas between the profiles, enabling a complete 3D surface map. Creating a 3D surface from the set of primary profiles provides a more comprehensive and representative depiction of the sample’s topography. Colored maps of the 3D reconstruction in an isometric view, with colors corresponding to local height variations, are shown in Figure 6.

From the 3D surface reconstruction, 3D surface parameters were determined using TalyMap software. The results are presented in Table 5.

The Sa parameter and the root mean square height (Sq) confirm the topographical variations previously observed in the 2D profiles. The lowest values were obtained for the top surface of sample S1-1 (Sa = 6.12 µm, Sq = 7.68 µm), while the highest were measured on the side surface of the same sample (Sa = 11.75 µm, Sq = 14.50 µm). For the side surface of sample S1-2, these values are comparable to S1-1 side (Sa = 10.98 µm, Sq = 14.14 µm), indicating a similar surface character.

The differences between the top and side surfaces are pronounced, with the top surface exhibiting approximately half the height variations compared to the side surfaces. This observation aligns with expectations from the FDM process, where the top surface, formed by the final extrusion paths, is smoother, while the side walls result from layer-by-layer deposition and exhibit a more stepped structure. The values of Sp (maximum peak height) and Sv (maximum valley depth) further support this trend. For S1-1, the top surface shows Sp = 23.7 µm and Sv = 32.2 µm, whereas the side surface reaches 39.6 µm and 45.2 µm, respectively. For the side of S1-2, these values are even higher (Sp = 36.8 µm, Sv = 66.2 µm), leading to a total profile height Sz = 102.99 µm, the highest among all analyzed surfaces. The large difference in Sv and Sz for S1-2 side suggests the presence of deeper valleys between filament paths, typical for printing at a 90° orientation, where the deposition direction promotes the formation of micro-indentations between layers.

The skewness parameter (Ssk) indicates whether a surface is dominated by peaks (Ssk > 0) or valleys (Ssk < 0). For the top surface of S1-1, Ssk = +0.343, reflecting a predominance of peaks, typical for top surfaces where the final extrusion layer can locally overflow, forming small protrusions. The side surface of S1-1 shows Ssk ≈ +0.042, suggesting an almost symmetric height distribution, i.e., a balanced contribution of peaks and valleys. For the side surface of S1-2, Ssk = −0.842, indicating a dominance of valleys. This demonstrates that printing at a 90° orientation creates deeper spaces between filament paths, consistent with the Sv and Sz parameters. The kurtosis parameter (Sku) for the top and side surfaces of S1-1 is 2.99 and 2.58, respectively, indicating a distribution close to normal (Sku ≈ 3). For the side surface of S1-2, Sku = 4.28, reflecting a more peaked distribution with pronounced individual peaks or valleys, indicating greater local variation in height.

### 3.4. Tensile Test

**Figure 7:**
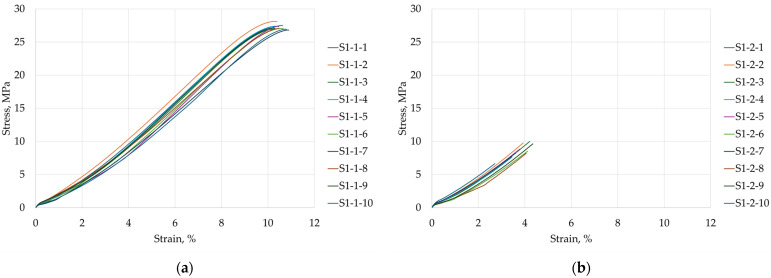
Stress–strain curves for samples: (**a**) S1-1, (**b**) S1-2.

In the next stage of the study, the samples were subjected to a static tensile test. During the test, stress and strain were recorded and calculated by the testing machine software based on the applied load, displacement, and the actual cross-sectional dimensions of the sample, which were determined from caliper measurements. The resulting stress–strain curves are presented in Figure 7.

Based on ten tensile tests, the mean tensile strength of the S1-1 samples was 27.24 MPa with a standard deviation of 0.38 MPa. For the S1-2 samples, the mean tensile strength was 8.58 MPa with a standard deviation of 1.03 MPa. During testing, S1-2 samples fractured in a brittle manner, showing no clear signs of material plasticization, whereas S1-1 samples exhibited the onset of a plastic region. The experimentally obtained values are lower than the manufacturer’s data, which indicate a tensile strength of approximately 35 MPa for 0° orientation and around 19 MPa for 90° orientation.

The mean tensile strength of the S1-2 samples (8.58 MPa) is approximately 68.5% lower than that of the S1-1 samples (27.24 MPa). According to the manufacturer’s data, the difference between the 90° (19 MPa) and 0° (35 MPa) orientations is about 45.7%. These discrepancies clearly indicate a strong anisotropy in the mechanical behavior of the steel-powder-reinforced thermoplastic composite, with tensile strength being significantly higher when the load is applied parallel to the deposited layers (0°) and much lower when applied perpendicular to the layers (90°), which is typical for FDM-printed parts due to interlayer bonding limitations.

### 3.5. Wettability Angle Measurement

**Figure 8:**
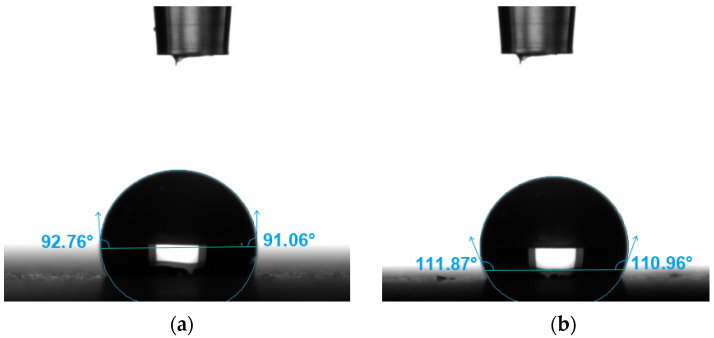
Examples of views of drops of demineralized water: (**a**) 0°, (**b**) 90°.

**Figure 9:**
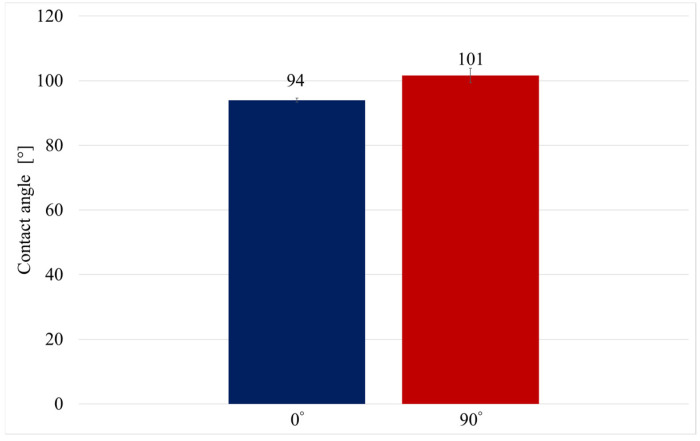
Average contact angle.

Figure 8 shows examples of obtained views of distilled water drops on the tested surfaces, and Figure 9 presents the average values of the obtained contact angles.

The average contact angle values with distilled water indicate the hydrophobic nature of both tested orientations. The 90° orientation exhibited a contact angle approximately 8% higher than the other orientation, indicating lower wettability. This difference can be attributed to the layer deposition pattern in FDM printing: surfaces oriented parallel to the build layers provide a more continuous and smoother interface for the liquid, enhancing adhesion, whereas surfaces perpendicular to the layers exhibit a stepped structure that reduces liquid spreading and increases the contact angle.

### 3.6. SEM Observations

**Figure 10:**
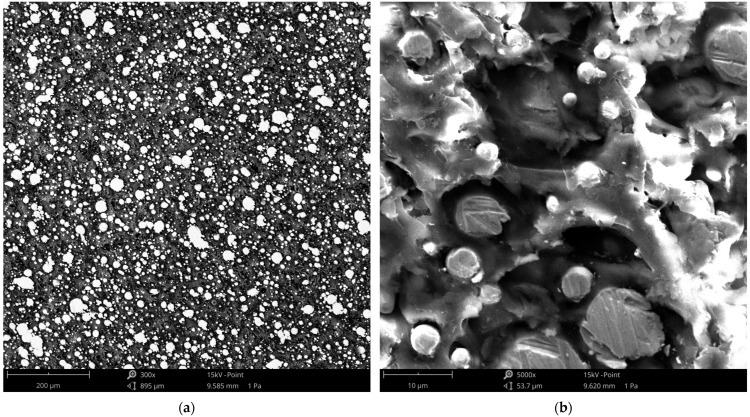
SEM view of sample S2-1 at magnification: (**a**) 300×, (**b**) 5000×.

Figure 10 shows the SEM images, illustrating the distribution of steel particles, voids, and characteristic craters within the polymer matrix at different magnifications.

SEM analysis of the steel-powder-reinforced composite revealed a heterogeneous microstructure characterized by the presence of steel particles of varying sizes embedded within a polymer matrix. At 300× magnification, the particles exhibited an oval shape and bright contrast, clearly distinguishable from the gray polymer matrix. The largest particles observed had diameters of approximately 40 µm. Dark regions corresponding to voids were also visible, indicating localized discontinuities in the material. At higher magnification (5000×), finer particles with diameters ranging from a few micrometers up to 10–15 µm were evident. Additionally, distinct oval-shaped craters could be seen, suggesting that some particles had detached from the matrix, likely during the extrusion or layer deposition process. These features imply incomplete particle embedding and the presence of micro-voids, which may reduce effective load transfer within the composite and partially explain the limited tensile strengthening observed in mechanical tests. The observed microstructural heterogeneity, including particle size distribution, voids, and surface craters, highlights the challenges of achieving uniform dispersion and strong interfacial bonding in highly filled metal-polymer FDM filaments.

## 4. Discussion

In composites, controlling the orientation and distribution of fillers, such as particles and fibers, remains a significant challenge. Random or improper fiber alignment exacerbates anisotropy in printed parts, as the material cannot fully exploit the reinforcing properties of the fibers. As a result, the overall strength and performance of the final components are reduced [17]. These issues become more pronounced in highly filled materials where increased stiffness and brittleness of the filament hinder its feeding, and reduced flexibility promotes buckling during extrusion. At the same time, even small variations in particle morphology at such high filler content can significantly increase the melt viscosity and hinder stable flow, which, combined with the rigidity of the particles, contributes to nozzle clogging. Process stability further depends on the properties of the filament, which must withstand feeding forces without breaking or being crushed, as well as on proper printing parameters that influence the risk of extrusion interruptions [18].

Despite numerous challenges associated with printing highly filled materials, such as increased filament brittleness, the risk of buckling, or nozzle clogging, it was possible to fabricate samples without noticeable signs of material discontinuities or flow issues in the print head (Figure 4). The sample thickness was higher than the nominal value (4 mm), exhibiting positive deviations of approximately 1–2%. Sample width depended on the print orientation: samples from series S1-1 (0°) were wider than S1-2 (90°), with a difference of about 0.10–0.12 mm, corresponding to 2–2.4% of the nominal value (Figure 3). Literature data [27] for ISO 527 samples show similar trends; for PLA, thickness deviations ranged from −0.75% to +5.25%, while width deviations ranged from +2.2% to +8.4%. In PLA—CF composites, thickness varied only slightly (+0.5% to +3.25%), whereas the width of samples printed at 90° was below the nominal value (−2.6%). Thus, the addition of carbon fibers had the greatest effect on the width of samples in the 90° orientation, while the thickness remained relatively close to the nominal value.

Literature data [28] indicate that nozzle size has a significant impact on dimensional accuracy and geometric deviations of FDM-printed components. Larger nozzle diameters tend to promote shape deviations and increase part dimensions relative to nominal values. In contrast, smaller nozzles allow for better control over part geometry and dimensions, as well as surface quality, reducing the staircase effect and surface roughness. In the context of the present study, printing with a 0.6 mm nozzle (larger than the standard 0.4 mm commonly recommended in the literature) may have contributed to slightly greater dimensional deviations in the samples, particularly in width and thickness, and to limited precision in geometric details.

The obtained results indicate that the orientation of the printed part influences both the geometric structure of the surface and the selected layer height as well as the nozzle diameter used for material extrusion (Table 4). Literature reports show that surface roughness is strongly correlated with layer height. For example, in the study [29] on PETG components, layer height was identified as the most significant factor affecting Ra. Specifically, thin layers of around 0.1 mm allow achieving Ra values of approximately 3 µm, whereas increasing the layer thickness leads to several-fold higher values. At the same time, studies on PLA components and fiber-reinforced polymers [30,31] demonstrate that surface roughness and waviness remain highly dependent on the toolpath direction and material type. Pure, unmodified PLA typically exhibits higher Ra values (3.5–10 µm), while polymer composites (PLA CF, ASA-Kevlar) allow for more stable, lower values in the range of 3–6 µm.

In the study [30], attention was drawn to pronounced differences between horizontal and vertical surfaces. In FDM-printed components, vertical surfaces are dominated by long-wavelength irregularities resulting from the distinct replication of individual layers along the *Z*-axis, leading to higher waviness and greater profile amplitudes. In contrast, top and bottom surfaces exhibit a more uniform character, where the main factor is the spacing between adjacent filament paths. The results obtained in the present study for the composite with steel powder filler indicate that these phenomena described in the literature are also characteristic of powder-filled materials. Samples printed in the 0° orientation (S1-1) exhibited the highest height-related parameters on the side surfaces (Pp = 34.1 µm, Pv = 37.4 µm, Pz = 71.5 µm), which is more than twice the value measured for the top surface of the same model (Pz ≈ 33 µm). This demonstrates that the micro-steps formed by successive layers are much more pronounced on vertical walls printed along the *Z*-axis. Similarly, the Pa and Pq parameters were significantly higher on the side surfaces, reflecting greater variability in the local surface height.

Comparison of the Psk and Pku parameters confirmed the influence of print orientation on the height distribution of surface irregularities. The top surface exhibited positive skewness (predominance of peaks), whereas the side surface of the sample printed at 90° showed negative skewness, indicating a predominance of valleys. This behavior is consistent with the observations reported by the researchers in Ref. [31], who also demonstrated that the way the measurement profile intersects layers and filament paths alters the distribution character; in fiber-reinforced composites, filament orientation produced a similar shift in skewness toward negative values. All analyzed surfaces had Pku < 3 (Table 4), indicating a flattened distribution without sharp peaks. In Ref. [31], such behavior was observed in both pure polymers and fiber-reinforced composites, where the sequential deposition of filament paths generated profiles with a limited number of prominent high points. The authors [31] interpret this effect as a result of “smoothing” local extrema through repeated nozzle passes and interference between adjacent paths, leading to the formation of characteristic ridges and valleys with gentle edges.

Analysis of the Psm parameter showed that the spatial distribution of profile features differs significantly between top and side surfaces. The Psm value of 0.394 mm measured on the top surface reflects larger spacing between filament paths, which may result from the use of a 0.6 mm nozzle. On the side surfaces, Psm values were considerably lower (0.166 mm for S1-1 and 0.121 mm for S1-2), confirming a more regular, layered topography. It is also noteworthy that the sample printed in the 90° orientation reproduced the layer structure with the highest repeatability, as evidenced by the lowest standard deviations in amplitude and spacing parameters. This phenomenon aligns with literature observations [31], which indicate that stiffer internal structures (characteristic of high infill densities or composites) promote more stable filament deposition and reduce local layer deformations. In the present case, the presence of steel powder may have contributed to increased local stiffness and more regular layer deposition, particularly in vertically oriented samples.

Analysis of the surface wettability of FDM-printed components indicates that both process parameters and build orientation can significantly influence the observed contact angle. In the literature on pure PLA [11], a pronounced anisotropy of the contact angle has been reported, related to the geometry of the filament paths and layer height: measurements conducted parallel to the filament path remain within the full wetting range (approximately 70–85°), whereas perpendicular measurements can reach values exceeding 105° when layer height or raster width increases. This phenomenon arises from directional roughness and the formation of micro-steps, which hinder droplet spreading and lead to partial wetting. Additionally, studies on PLA composites with lignocellulosic fillers [32] indicate that the presence of a filler phase can significantly alter the surface characteristics. For PLA/ROA composites, the material becomes noticeably more hydrophilic (70–81°), with wettability further enhanced at higher printing speeds, which is attributed to reduced time for filament trace fixation and smoothing of micro-irregularities.

In the present study, the results for the steel powder-filled composite show a different trend: both tested orientations exhibited contact angles above 90°, indicating a clearly hydrophobic behavior, in contrast to polar polymer-based composites. The approximately 8% difference between orientations confirms that the build direction in FDM is a key factor influencing wettability, consistent with the mechanisms described in [11]. Surfaces printed in the 0° orientation exhibited lower contact angles because they form a more continuous plane parallel to the filament paths, promoting liquid adhesion. In contrast, in the 90° orientation, where the droplet interacts with a pronounced stepped structure resulting from vertical layer deposition, the contact angle increased to over 100°, correlating with the observed contact angle anisotropy in printed structures with high layer heights and distinct directional micro-roughness. The obtained results also suggest that the presence of steel powder does not reduce the contact angle; rather, combined with the characteristic surface topography, it contributes to increased hydrophobicity.

Tensile testing of the steel-powder-reinforced composite revealed a pronounced anisotropy in mechanical behavior. Samples printed in the 0° orientation (S1-1) exhibited a mean tensile strength of 27.24 MPa and showed the onset of plastic deformation, whereas samples printed at 90° (S1-2) had a mean tensile strength of 8.58 MPa and fractured in a brittle manner, with no visible plasticity. The difference between orientations is approximately 68.5%, highlighting the critical influence of layer deposition direction, which is typical for FDM parts due to limited interlayer bonding. As noted previously, the filament consists of 316 L steel powder embedded in a proprietary thermoplastic polymer, likely a high-temperature material with properties similar to PETG, based on its extrusion temperature (~245 °C) and processing characteristics. Comparison with literature data for PETG [33] shows that tensile strengths above 30 MPa are achievable at 100% infill for flat 0° prints, indicating that the steel-filled composite did not exhibit material strengthening. SEM observations (Figure 10) suggest that this limitation may result from voids or gaps in the material, possibly caused by the detachment or loss of individual steel particles during extrusion, which reduces effective load transfer within the matrix.

## 5. Conclusions

The results allowed for drawing the following conclusions:

- The build orientation significantly influences the surface structure, topography, and tensile strength of the thermoplastic composite with steel powder filler;
- Top-layer surfaces exhibit more uniform topography compared to the side-layer surfaces, which results from the nature of material deposition during the FDM process;
- Side-layer surfaces are characterized by higher height parameters (Pp, Pv, Pz, Pt) and a more heterogeneous topography, which is typical of the stair-step effect inherent to layer-by-layer printing;
- Tensile strength is markedly higher in the 0° orientation (average 27.24 MPa) compared to the 90° orientation (average 8.58 MPa), confirming the pronounced mechanical anisotropy arising from the build direction;
- Both orientations exhibit hydrophobic behavior, with surfaces perpendicular to the build layers (90°) being more hydrophobic due to their stepped topography, while parallel layers show relatively higher wettability.

The tested composite contained an exceptionally high loading of 95 wt.% steel powder, with only 5 wt.% thermoplastic matrix. Despite this extreme filler content, the material was successfully processed via FDM 3D printing, which is extraordinary given that such high particle loadings typically lead to poor flow, nozzle clogging, and compromised print quality. Additionally, the material is resistant to environmental conditions; however, the high steel content significantly increases the weight of the printed models due to the higher density of the composite.

## Figures and Tables

**Table 1:** Material properties of AIB METAL [21].

Parameter	Test Method	Value
Tensile strength XY	ISO 527-1 [22]	35 +/− 2 MPa
Tensile strength ZX	ISO 527-1	19 +/− 2 MPa
Elastic modulus	ISO 527-1	1.5 +/− 0.1 GPa
Tensile yield strain	ISO 527-1	5.1 +/− 0.1%
Flexural strength	ISO 178 [23]	66 +/− 2 MPa
Flexural deflection	ISO 178	9.0 +/− 0.1 mm
Charpy notched impact strength	ISO 179-1 [24]	6 +/− 1 kJ/m^2^
Density	ISO 1183 [25]	4 g/cm^3^
Glass transition temperature	ISO 11357 [26]	81 °C

**Table 2:** Additive manufacturing parameters.

Parameter	Value	Unit
Layer Height	0.12	mm
Wall loops	2	-
Infill pattern	Linear	-
Infill density	100	%
Nozzle temperature	245	°C
Bed temperature	80	°C
Print speed	80	mm/s
First layer height	0.30	mm

**Table 3:** Sample designations.

Sample Designation	Number of Repetitions	Orientation
S1-1	10	0°
S1-2	10	90°
S2-1	5	0°
S2-2	5	90°

**Table 4:** Primary profile parameters.

Parameter	Mean Value			Standard Deviation		
	S1-1 Top	S1-1 Side	S1-2 Side	S1-1 Top	S1-1 Side	S1-2 Side
Pp, µm	17.234	34.101	24.252	3.971	2.677	3.678
Pv, µm	15.729	37.383	27.329	3.392	3.715	1.588
Pz, µm	32.963	71.484	51.581	6.118	4.138	3.799
Pc, µm	14.465	31.328	29.918	3.740	1.217	0.667
Pt, µm	32.963	71.484	51.581	6.118	4.138	3.799
Pa, µm	5.087	11.685	8.546	1.020	0.477	0.223
Pq, µm	6.237	14.400	10.399	1.181	0.562	0.304
Psk	0.347	0.052	−0.341	0.354	0.130	0.087
Pku	2.831	2.541	2.671	0.578	0.133	0.119
Psm, mm	0.394	0.166	0.121	0.129	0.015	0.004

**Table 5:** Values of parameters of surface topography.

Parameter	Value		
	S1-1 Top	S1-1 Side	S1-2 Side
Sq, µm	7.682	14.497	14.141
Ssk	0.343	0.042	−0.842
Sku	2.993	2.579	4.280
Sp, µm	23.715	39.647	36.820
Sv, µm	32.170	45.182	66.169
Sz, µm	55.885	84.829	102.990
Sa, µm	6.124	11.749	10.983

## Data Availability

The original contributions presented in this study are included in the article. Further inquiries can be directed to the corresponding author.
